# Prostaglandin E2 mediates sensory nerve regulation of bone homeostasis

**DOI:** 10.1038/s41467-018-08097-7

**Published:** 2019-01-14

**Authors:** Hao Chen, Bo Hu, Xiao Lv, Shouan Zhu, Gehua Zhen, Mei Wan, Amit Jain, Bo Gao, Yu Chai, Mi Yang, Xiao Wang, Ruoxian Deng, Lei Wang, Yong Cao, Shuangfei Ni, Shen Liu, Wen Yuan, Huajiang Chen, Xinzhong Dong, Yun Guan, Huilin Yang, Xu Cao

**Affiliations:** 1 Department of Orthopaedic Surgery, The Johns Hopkins University, Baltimore, MD 21205 USA; 2 Department of Orthopaedic Surgery, First Affiliated Hospital of Soochow University, Suzhou, Jiangsu 215000 P. R. China; 3 Section of Spine Surgery, Department of Orthopaedics, Changzheng Hospital, Second Military Medical University, Shanghai, 200433 P. R. China; 4 Howard Hughes Medical Institute and The Solomon H. Snyder Department of Neuroscience, Center for Sensory Biology, The Johns Hopkins University School of Medicine, Baltimore, MD 21205 USA; 5 Department of Anesthesiology and Critical Care Medicine, The Johns Hopkins University School of Medicine, Baltimore, MD 21205 USA

## Abstract

Whether sensory nerve can sense bone density or metabolic activity to control bone homeostasis is unknown. Here we found prostaglandin E2 (PGE2) secreted by osteoblastic cells activates PGE2 receptor 4 (*EP4*) in sensory nerves to regulate bone formation by inhibiting sympathetic activity through the central nervous system. PGE2 secreted by osteoblasts increases when bone density decreases as demonstrated in osteoporotic animal models. Ablation of sensory nerves erodes the skeletal integrity. Specifically, knockout of the *EP4* gene in the sensory nerves or cyclooxygenase-2 (*COX2*) in the osteoblastic cells significantly reduces bone volume in adult mice. Sympathetic tone is increased in sensory denervation models, and propranolol, a β2-adrenergic antagonist, rescues bone loss. Furthermore, injection of SW033291, a small molecule to increase PGE2 level locally, significantly boostes bone formation, whereas the effect is obstructed in *EP4* knockout mice. Thus, we show that PGE2 mediates sensory nerve to control bone homeostasis and promote regeneration.

## Abstract (web summary)

Bone is innervated, and its turnover is affected by sympathetic nerve activity. Here, the authors show that prostaglandin E2, secreted by osteoblasts, activates the EP4 receptor on sensory nerves, inhibiting sympathetic nerve activity and modulating bone formation in mice.

## Introduction

Sensory nerves are innervated in peripheral tissues, including skin, joint, respiratory, and gastrointestinal tissues, to sense stimuli inside or outside the body, such as pain, temperature, odors, and tastes^1,2^. The signals collected from sensory nerve endings are processed in the central nervous system to initiate physiological responses. Bone is the largest organ, accounting for more than 80% of body weight. Bone is also an endocrine organ that regulates calcium and mineral metabolism, glucose, fatty acids, and even cancer metastasis by interacting with other tissues^3–5^. The skeleton has abundant sensory and sympathetic innervations^6–9^ and interacts with the central nervous system^10,11^. Sympathetic nerves induce catabolic activity in bone through serotonin and cAMP-response element binding protein (CREB) signaling in the hypothalamus^10,12,13^. Specific deletion of sensory nerves in bone impairs bone mass accrual^14,15^. Patients with sensory nerve malfunction or loss have an increased bone fracture rate and significantly diminished post-injury bone regeneration^16,17^. These observations indicate that sensory nerves sense changes in bone density, mechanical stress, and metabolic activity to control bone homeostasis.

Based on the evidence, one or more molecules should transmit signals of changes in bone to sensory nerve fibers. Cyclooxygenase activity and prostaglandins are known to mediate skeletal metabolism and inflammation^18,19^. Among prostaglandins, prostaglandin E2 (PGE2) is a multifunctional molecule whose production is controlled by the limiting enzyme cyclooxygenase (COX)^18^. Evidence shows that PGE2 can elicit primary pain and prolong nociceptor sensitization^20,21^. Non-steroidal anti-inflammatory drugs and COX2 selective inhibitors are the current major medications to treat musculoskeletal pain^22^. A multicenter study revealed that COX2 selective inhibitor is associated with lower bone mineral density (BMD) in men; whereas, in postmenopausal women it promotes BMD^23^, implicating PGE2 in the regulation of bone.

The 15-hydroxyprostaglandin dehydrogenase gene *(HPGD)* encodes a NAD^+^-dependent 15-hydroxyprostaglandin dehydrogenase (*15-PGDH*), which catalyzes PGE2. Mutation of this gene impairs the degradation of PGE2^24^. *HPGD* mutant mice showed an increased PGE2 level in vivo, which can effectively promote regeneration in different tissues^25–27^. Interestingly, patients with *HPGD* mutation have presented with subperiosteal new bone formation^28^. PGE2 is also potent in stimulating bone formation, and its bone anabolic effect is believed to be through its receptor EP4 in the osteoblasts^18,29,30^. However, conditional knockout of the prostaglandin E receptor 4 gene (*EP4*) in osteoblastic cells did not impair bone density, implying that the bone formation effect of PGE2 does not act through osteoblasts^31^. In pathological conditions of bone loss during aging or after menopause, the impaired function of sensory nerves and elevated PGE2 level appear simultaneously^32,33^. Thus, PGE2-induced pain may reflect its activation of sensory nerves to transmit a signal of bone density to maintain bone homeostasis.

Here in this study, we report that bone density regulates the level of PGE2 secreted by osteoblasts. Deletion of *EP4* in sensory nerves or *COX2* in osteoblasts significantly decreases bone mass. Elevation of PGE2 by inhibiting 15-PDGH promotes bone regeneration. PGE2 regulates osteoblast bone formation by activation of sensory nerves in a sympathetic nerve feedback manner.

## Results

### Sensory denervation reduces osteoblastic bone formation

**Fig. 1:**
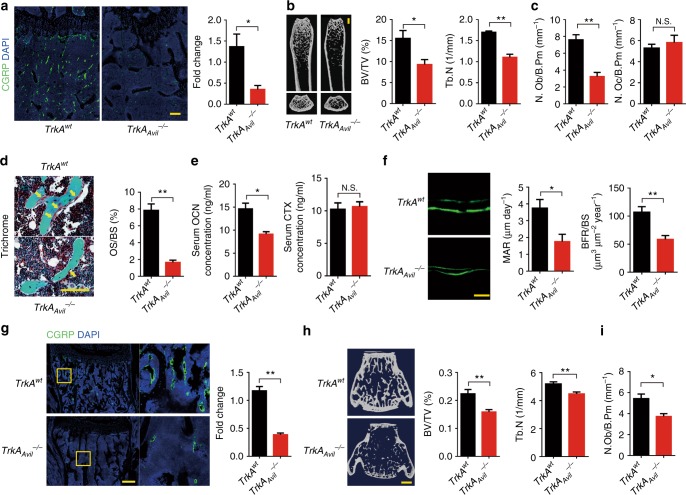
Osteoblastic bone formation is reduced without sensory nerve innervation. **a** Representative images of immunofluorescence staining and quantitative analysis of the CGRP^+^ sensory nerves (green) in the femurs of 12-week-old *TrkA*^*wt*^ and *TrkA*_*Avil*_^*−/−*^ mice. DAPI stains nuclei blue. Scale bar: 100 μm. **b** Representative micro-computed tomography (μCT) images of femurs from 12-week-old male *TrkA*^*wt*^ and *TrkA*_*Avil*_^*−/−*^ mice. Quantitative analysis of trabecular bone fraction (Tb. BV/TV) and trabecular number (Tb. N). Scale bar: 1 mm. **c** Histomorphological analysis of osteoblast (N.Ob/B.Pm) and osteoclast (N.Oc/B.Pm) numbers on the trabecular bone surface of femurs of 12-week-old *TrkA*^*wt*^ and *TrkA*_*Avil*_^*−/−*^ mice. **d** Trichrome staining and quantitative analysis of osteoid surface per bone surface (OS/BS) in femoral bone tissue from 12-week-old *TrkA*^*wt*^ and *TrkA*_*Avil*_^*−/−*^ mice. Scale bar, 50 μm. **e** ELISA analysis of serum OCN and CTX levels in 12-week-old *TrkA*^*wt*^ and *TrkA*_*Avil*_^*−/−*^ mice. **f** Representative images of calcein double labeling of trabecular bone of femurs with quantification of mineral apposition rate and bone formation rate in 12-week-old *TrkA*^*wt*^ and *TrkA*_*Avil*_^*−/−*^ mice. Scale bar, 20 μm. **g** Representative images of immunofluorescence staining and quantitative analysis of the CGRP^+^ sensory nerves (green) in the vertebrae of 12-week-old *TrkA*^*wt*^ and *TrkA*_*Avil*_^*−/−*^ mice. DAPI stains nuclei blue. Scale bar: 100 μm. **h** Representative μCT images of vertebra from 12-week-old *TrkA*^*wt*^ and *TrkA*_*Avil*_^*−/−*^ mice. Quantitative analysis of trabecular bone fraction (Tb. BV/TV) and trabecular number (Tb. N). Scale bar: 1 mm. **i** Histomorphological analysis of osteoblast (N.Ob/B.Pm) numbers on the trabecular bone surface of 12-week-old *TrkA*^*wt*^ and *TrkA*_*Avil*_^*−/−*^ mice vertebra. *N* ≥ 5 per group. *P < 0.05, **P < 0.01 and N.S. means not significant. (Student *t*-test)

To investigate the effect of sensory nerve in bone, we created a sensory denervation mouse model (*TrkA*_*Avil*_^*−/−*^*)* by crossing sensory nerve-specific cre (Advillin-cre) mice with nerve growth factor (NGF) receptor *TrkA* floxed (*TrkA*^*wt*^) mice. Quantitative polymerase chain reaction (qPCR) and immunofluorescent staining of TrkA in the dorsal root ganglion (DRG) neurons and the other tissues isolated from the *TrkA*_*Avil*_^*−/−*^ mice validated the knockout efficiency and specificity of the *TrkA* gene in the *TrkA*_*Avil*_^*−/−*^ mice (Supplementary Figure 1). Furthermore, immunostaining of femur sections showed that most calcitonin gene-related peptide (CGRP)^+^ sensory nerve fibers were eliminated in the *TrkA*_*Avil*_^*−/−*^ mice (Fig. 1a). Significant bone loss was observed in 12-week-old *TrkA*_*Avil*_^*−/−*^ mice relative to their wild-type (WT) littermates in μCT analysis (Fig. 1b), while no significant bone volume decrease was found of 4-week-old age (Supplementary Figure 2), indicating an essential role of sensory nerve for bone homeostasis in adults. The number of osteocalcin^+^ osteoblasts was significantly lower in *TrkA*_*Avil*_^*−/−*^ mice relative to their WT littermates; whereas, the number of tartaric acidic phosphatase (TRAP)^+^ osteoclasts was not different (Fig. 1c). Trichrome staining showed decreased osteoid in *TrkA*_*Avil*_^*−/−*^ mice (Fig. 1d). Accordingly, the serum level of osteocalcin, a marker of osteoblastic bone formation, was significantly lower, and the level of osteoclast bone resorption marker carboxy-terminal collagen crosslinks (CTX) was not different in *TrkA*_*Avil*_^*−/−*^ mice (Fig. 1e). Calcein double labeling confirmed the reduced bone formation and mineral apposition rate (Fig. 1f). We also evaluated the sensory innervations and bone architecture in the vertebrae of *TrkA*_*Avil*_^*−/−*^ mice, and similar results were observed (Fig. 1g–i and Supplementary Figure 3, A–C). These results indicate that sensory nerve regulates osteoblastic bone formation in adult mice.

**Fig. 2:**
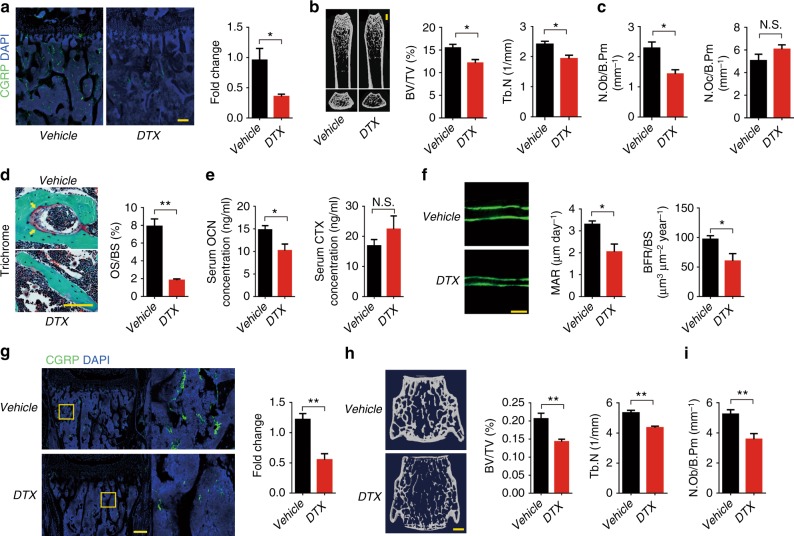
Osteoblastic bone formation is blunted after sensory denervation. **a** Representative images of immunofluorescence staining and quantitative analysis of the CGRP^+^ sensory nerves in the femurs of 8-week-old *iDTR*_*Avil*_^*+/−*^ mice injected with vehicle or 1 ug per kg per day DTX 3 time a week for four consecutive weeks. Scale bar: 100 μm. **b** Representative μCT images of femurs from *iDTR*_*Avil*_^*+/−*^ mice injected with vehicle or DTX. Quantitative analysis of trabecular bone fraction and trabecular number. Scale bar: 1 mm. **c** Histomorphological analysis of the osteoblast (N.Ob/B.Pm) and osteoclast (N.Oc/B.Pm) numbers on the trabecular bone surface of femurs of *iDTR*_*Avil*_^*+/−*^ mice injected with vehicle or DTX. **d** Representative trichrome staining and quantitative analysis of OS/BS in femoral bone tissue from *iDTR*_*Avil*_^*+/−*^ mice injected with vehicle or DTX. Scale bar, 50 μm. **e** ELISA analysis of serum OCN and CTX levels in *iDTR*_*Avil*_^*+/−*^ mice injected with vehicle or DTX. **f** Representative images of calcein double labeling of femoral trabecular bone with quantification of MAR and BFR in *iDTR*_*Avil*_^*+/−*^ mice injected with vehicle or DTX. Scale bar, 20 μm. **g** Representative images of immunofluorescence staining and quantitative analysis of the CGRP^+^ sensory nerves (green) in the vertebra of *iDTR*_*Avil*_^*+/−*^ mice injected with vehicle or DTX. DAPI stains nuclei blue. Scale bar: 100 μm. **h** Representative μCT images of vertebrae from *iDTR*_*Avil*_^*+/−*^ mice injected with vehicle or DTX. Quantitative analysis of trabecular bone fraction (Tb. BV/TV) and trabecular number (Tb. N). Scale bar: 1 mm. **i** Histomorphological analysis of osteoblast (N.Ob/B.Pm) numbers on the trabecular bone surface of 12-week-old *TrkA*^*wt*^ and *TrkA*_*Avil*_^*−/−*^ mice vertebra. *N* ≥ 5 per group. *P < 0.05, **P < 0.01 and N.S. means not significant. (Student *t*-test)

To examine whether sensory nerves maintain bone homeostasis through bone remodeling in adult mice, we established inducible sensory denervation in *iDTR*_*Avil*_^+/−^ mice by crossing Advillin-cre mice with *iDTR*^*wt*^ mice. Sensory denervation was effectively induced in adult *iDTR*_*Avil*_^*fl/−*^ mice by injection of 1 ug per kg diphtheria toxin (DTX) three times a week for four weeks (Fig. 2a). Significant bone loss was observed using μCT (Fig. 2b). Similarly, the number of osteoblasts, amount of osteoid, and serum osteocalcin level were significantly decreased; whereas, the number of osteoclasts and serum CTX level were unchanged relative to the vehicle group (Fig. 2c, e). The calcein double-labeling experiment confirmed that bone formation and mineral apposition rate were reduced (Fig. 2f). Moreover, sensory innervations and bone mass also decreased in the vertebrae of *iDTR*_*Avil*_^*fl/−*^ mice injected with DTX relative to the vehicle-treated mice (Fig. 2g, i and Supplementary Figure 3, D–E). Because neural changes other than those in the sensory nervous system can affect bone metabolism indirectly, we performed pole tests and grip strength tests with *TrkA*^*−/−*^ mice and *iDTR*_*Avil*_^*fl/-*^ mice injected with DTX. No motor neural activity was influenced in these two mouse models (Supplementary Figure 4). Thus, sensory nerve regulates bone homeostasis through osteoblasts during bone remodeling.

### Knockout of EP4 receptor in sensory nerve induces bone loss

**Fig. 3:**
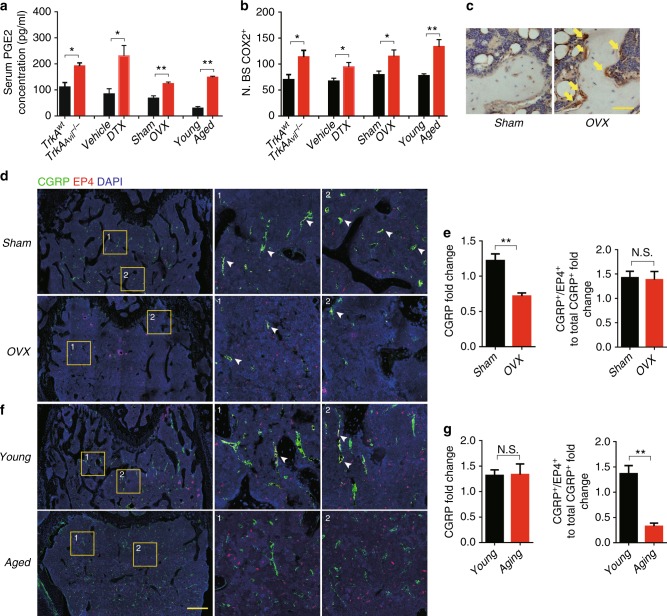
Elevated PGE2 secretion from osteoblasts with sensory nerve dysfunction. **a** ELISA analysis of serum PGE2 levels in mice with different treatments, including *TrkA*^*wt*^ and *TrkA*_*Avil*_^*−/−*^ mice; *iDTR*_Avil_^*+/−*^ mice injected with vehicle or DTX; young (2-month-old) and aged (12-month-old) mice; mice with sham or ovariectomy surgery (OVX) for 8 weeks. **b** Quantitative analysis of the *COX2*^+^ cells (brown) on trabecular surface of femoral bone from different mice models, including *TrkA*^*wt*^ and *TrkA*_*Avil*_^*−/−*^ mice; *iDTR*_Avil_^*+/−*^ mice injected with vehicle or DTX; young (2-month-old) and aged (12-month-old) mice; and mice with sham or ovariectomy surgery (OVX) for 8 weeks. **c** Representative images of immunostaining of the *COX2*^+^ cells (brown) on trabecular surface of femoral bone from mice with sham or OVX for 8 weeks. Scale bar, 20 μm. **d**, **e** Representative double-immunofluorescent staining imaged and quantitative analysis of *EP4* (red) and CGRP (green) in femurs from mice underwent sham or OVX surgery. Scale bar: 100 μm. **f**, **g** Representative double-immunofluorescent staining images and quantitative analysis of *EP4* (red) and CGRP (green) in femurs from young and aged mice. Scale bar: 100 μm. *N* ≥ 5 per group. *P < 0.05, **P < 0.01 and N.S. means not significant. (Student *t*-test)

**Fig. 4:**
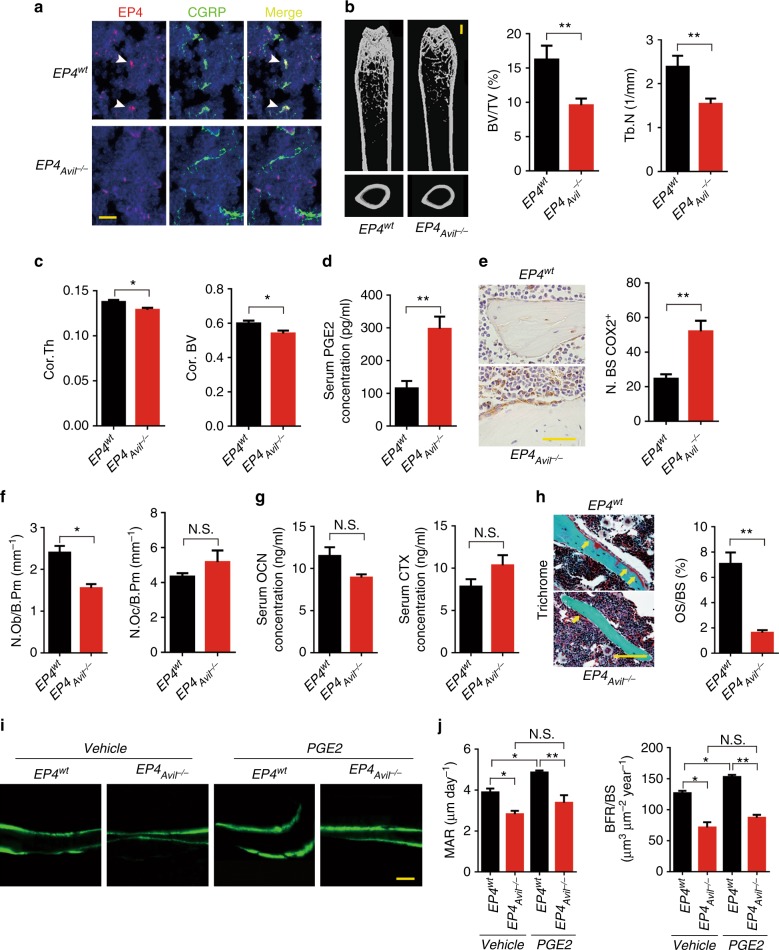
Deletion of PGE2 receptor EP4 in sensory nerve results in bone loss. **a** Double-immunofluorescence images of femoral bone sections from 12-week-old *EP4*^*wt*^ or *EP4*_*Avil*_^*−/−*^ mice using antibodies against *EP4* (red) and CGRP (green). DAPI stains nuclei blue. Scale bar, 50 μm. **b**, **c** Representative μCT images of femurs from 12-week-old *EP4*^*wt*^ and *EP4*_*Avil*_^*−/−*^ mice. Quantitative analysis of trabecular bone fraction (Tb. BV/TV), trabecular number (Tb. N), cortical thickness (Ct. Th), and cortical bone volume (Cor. BV). **d** ELISA analysis of serum PGE2 level in 12-week-old *EP4*^*wt*^ and *EP4*_*Avil*_^*−/−*^ mice. **e** Representative images of immunostaining and quantitative analysis of the *COX2*^+^ cells (in brown) on trabecular bone surface of femoral bone from 12-week-old *EP4*^*wt*^ and *EP4*_*Avil*_^*−/−*^ mice. Scale bar, 20 μm. **f** Histomorphological analysis of osteoblast (N.Ob/B.Pm) and osteoclast (N.Oc/B.Pm) numbers on the trabecular bone surface of femurs of 12-week-old *EP4*^*wt*^ and *EP4*_*Avil*_^*−/−*^ mice. **g** ELISA analysis of serum OCN and CTX levels in 12-week-old *EP4*^*wt*^ and *EP4*_*Avil*_^*−/−*^ mice. **h** Representative trichrome staining and quantitative analysis of osteoid surface per bone surface (OS/BS) in femoral bone tissue of 12-week-old *EP4*^*wt*^ and *EP4*_*Avil*_^*−/−*^ mice. Scale bar, 50 μm. **i**, **j** Ten-week-old *EP4*^*wt*^ and *EP4*_*Avil*_^*−/−*^ mice were injected with vehicle or 3 mg per kg per day PGE2 for 3 consecutive days, and bone samples were harvested 12 days after injection. Calcein was injected 5 days and 1 day before sacrifice. Representative images of calcein double labeling of femoral trabecular bone with quantification of mineral apposition rate (MAR) and bone formation rate (BFR). Scale bar, 20 μm. *N* ≥ 5 per group. *P < 0.05, **P < 0.01 and N.S. means not significant. (Student *t-*test, except **j** with ANOVA)

Because PGE2 is known to stimulate osteoblastic bone formation, we measured PGE2 levels in the serum of both global and inducible sensory denervation mice. Interestingly, PGE2 levels increased significantly in all the denervation mouse models (Fig. 3a). The results prompted us to examine whether PGE2 mediates sensory nerve in regulation of osteoblast bone formation. We found that bone density was negatively correlated with PGE2 levels, and that PGE2 levels increased in aged or the other osteoporotic mice (Fig. 3a). Immunohistochemical analysis also showed that expression of COX2 in femur osteoblasts, the PGE2 production-limiting enzyme, increased in the sensory denervation OVX and aged mice (Fig. 3b, c). As EP4 is the primary receptor of PGE2 for bone formation^34^, we further co-immunostained of EP4 or CGRP in OVX and aged mice femurs. In OVX mice, we observed a significant reduction of CGRP^+^ sensory fibers two weeks post OVX surgery (Fig. 3d, e). Interestingly, loss of EP4 expression in the sensory fibers of aged mouse bone marrow with no significant decrease of the CGRP^+^ nerve fibers (Fig. 3f, g). We then induced ablation of *EP4* in sensory nerves using adult *EP4*_*Avil*_^*−/−*^ mice by crossing Advillin-cre mice with *EP4*^*wt*^ mice to validate of EP4 function in sensory nerves. qPCR analysis and immunostaining of *EP4* confirmed that *EP4* deletion was specifically in nerve (Supplementary Figure 5). Co-immunofluorescent staining of EP4 with CGRP showed that EP4 was expressed in bone sensory nerves, confirming that *EP4* was efficiently deleted from sensory nerves in the bone marrow of *EP4*_*Avil*_^*−/−*^ mice (Fig. 4a).

Both trabecular bone and cortical bone decreased significantly in 12-week *EP4*-ablated mice (Fig. 4b, c, Supplementary Figure 6). Pole tests and grip strength tests showed no changes in motor neural activity (Supplementary Figure 7). PGE2 levels in the serum and COX2 expression in osteoblasts increased (Fig. 4d, e), suggesting a compensatory increase of the ligand PGE2 in response to *EP4* knockout in sensory nerves. Similar to the two sensory denervation models, in *EP4*_*Avil*_^*−/−*^ mice, the number of osteoblasts decreased (but the serum osteocalcin level showed no statistical significance), with no changes in osteoclast number or bone degradation marker CTX (Fig. 4f, g). Importantly, PGE2 induced significant bone formation in wild-type mice was abolished in *EP4*_*Avil*_^*−/−*^ mice, as demonstrated in trichrome and double-labeling experiments (Fig. 3h, j). As EP4 expression is also known to express in osteoblasts, osteoblastic cell-specific knockout *EP4* mice were generated by crossing *EP4*^*wt*^ mice with osteocalcin-cre (OC-cre) mice (Supplementary Figure 8, A). However, μCT analysis did not reveal significant bone volume change in *EP4*_*OC*_^*−/−*^ mice (Supplementary Figure 8, B). These results show that PGE2-induced osteoblastic bone formation is signaled through EP4 in the sensory nerves.

### PGE2 mediates sensory nerve induced osteogenesis

**Fig. 5:**
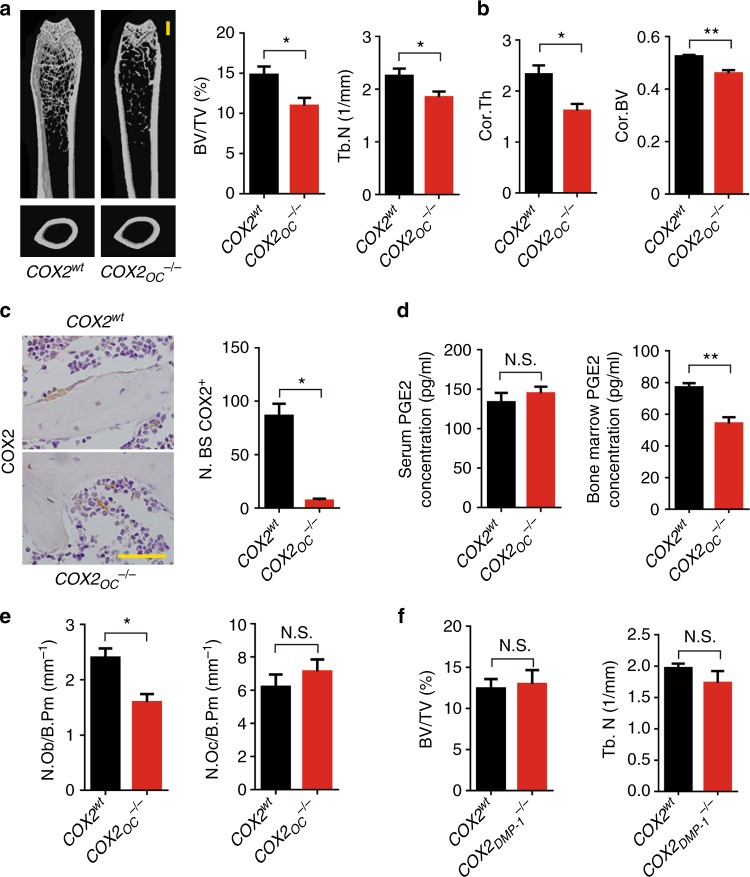
Ablation of COX2 in osteoblasts leads to reduced bone formation. **a** Representative μCT images of the femurs of 12-week-old *COX2*^*wt*^ and *COX2*_*OC*_^*−/−*^ mice. Quantitative analysis of the trabecular bone fraction (Tb. BV/TV) and trabecular number (Tb. N). Scale bar: 1 mm. **b** Quantitative analysis of cortical thickness (Ct. Th) and cortical bone volume (Cor. BV). **c** Representative images of immunostaining and quantitative analysis of the *COX2*^+^ cells (brown) on trabecular bone surface of femoral bone from 12-week-old *COX2*^*wt*^ and *COX2*_*OC*_^*−/−*^ mice. Scale bar, 20 μm. **d** ELISA analysis of the serum and bone marrow PGE2 in 12-week-old *COX2*^*wt*^ and *COX2*_*OC*_^*−/−*^ mice. **e** Histomorphological analysis of osteoblast (N.Ob/B.Pm) and osteoclast (N.Oc/B.Pm) numbers on the trabecular bone surface of 12-week-old *COX2*^*wt*^ and *COX2*_*OC*_^*−/−*^ mice. **f** Quantitative analysis of the trabecular bone fraction (Tb. BV/TV) and trabecular number (Tb. N) of the femurs from *COX2*^*wt*^ and *COX*_*DMP-1*_^*−/−*^ mice. *N* ≥ 5 per group. *P < 0.05, **P < 0.01 and N.S. means not significant. (Student *t-*test)

To examine whether PGE2 is secreted primarily by osteoblastic cells for sensory nerve regulation of bone, we further generated conditional knockout *COX2* mice in the osteoblastic cells (*COX2*_*OC*_^*−/−*^) by crossing *COX2*^*wt*^ mice with OC-cre mice to eliminate PGE2 secretion by osteoblastic cells. Pole tests and grip strength tests showed no effect on motor activity, indicating that knockout of *COX2* did not affect global neural activity (Supplementary Figure 9). As in *EP4* knockout mice, trabecular and cortical bone decreased while body weight remained unchanged over time in *COX2*_*OC*_^*−/−*^ mice relative to their *COX2*^*wt*^ littermates (Fig. 5a, b, Supplementary Figure 10). COX2 staining of the mouse femurs confirmed the expression of COX2 and its ablation in osteoblasts (Fig. 5c). Interestingly, CGRP^+^ nerve fibers were located in the active bone remodeling areas with Ocn^+^ osteoblasts in co-immunostaining (Supplementary Figure 11). This suggests that PGE2 secreted by osteoblastic cells in active bone remodeling sites is essential because the bone marrow PGE2, instead of the serum PGE2 levels, was significantly different between these two groups of mice (Fig. 5d). Thus, PGE2 secreted by osteoblastic cells in active bone remodeling sites mediates sensory nerve–stimulated bone formation. Again, the number of osteoblasts and serum osteocalcin level decreased significantly, whereas TRAP^+^ osteoclast number and serum CTX level were unchanged (Fig. 5e).

We also deleted *COX2* in osteocytes embedded in the bone matrix from terminal differentiation of osteoblasts to examine PGE2 in the osteoblastic bone-forming microenvironment essential for sensory nerve-induced osteogenesis. Crossing *DMP1*-cre mice with *COX2*^*wt*^ mice generates *COX2*_*DMP1*_^*−/−*^ mice to eliminate PGE2 secretion by osteocytes. Interestingly, bone phenotype was unchanged in *COX2*_*DMP1*_^*−/−*^ mice relative to their *COX2*^*wt*^ littermates (Fig. 5f). Taken together, these results show that PGE2 in the active bone-forming microenvironment, largely secreted by osteoblasts, mediates sensory nerve-regulated osteoblastic bone formation.

### PGE2 induces hypothalamic CREB signaling for osteogenesis

**Fig. 6:**
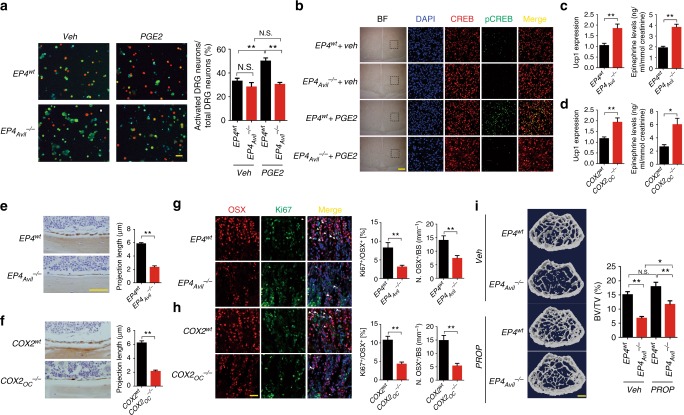
PGE2 stimulates hypothalamic CREB signaling through sensory nerve. **a** Representative images of DRG neurons isolated from *EP4*^*wt*^ and *EP4*_*Avil*_^*−/−*^ mice pre-incubated with vehicle or 10 μM PGE2 for 5 min, and subsequently treated with calcium imaging buffer (with calcium loaded). The red dots represent activated DRG neurons, and the green dots represent resting DRG neurons. Scale bar, 50 μm. Quantitative analysis was performed with results from three independent assays. **b** Double-immunofluorescence images of hypothalamus tissue sections from 12-week-old *EP4*^*wt*^ or *EP4*_*Avil*_^*−/−*^ mice with vehicle or 3 mg per kg PGE2 treatment for 6 h using antibodies against CREB (red) and p-CREB (green). DAPI stains nuclei blue. Scale bar, 20 μm. **c** qRT-PCR analysis of *UCP1* expression in adipose tissue and ELISA evaluation of epinephrine level of the serum from *EP4*^*wt*^ and *EP4*_*Avil*_^*−/−*^ mice. **d** qRT-PCR analysis of *UCP1* expression in adipose tissue and ELISA evaluation of epinephrine level of the serum from *COX2*^*wt*^ and *COX2*_*OC*_^*−/−*^ mice. **e**, **f** Representative images of immunostaining of the femoral bone tissue sections from *EP4*^*wt*^ and *EP4*_*Avil*_^*−/−*^ and *COX2*^*wt*^ and *COX2*_*OC*_^*−/−*^ mice with antibody against OCN. Scale bar, 50 μm. Projection length of the OCN^+^ lining cells was measured. **g**, **h** Double-immunofluorescence images of femoral bone tissue sections from 12-week-old *COX2*^*wt*^ and *COX2*_*OC*_^*−/−*^ and *EP4*^*wt*^ and *EP4*_*Avil*_^*−/−*^ mice using antibodies against OSX (red) and Ki67 (green). DAPI stains nuclei blue. Scale bar, 20 μm. Percentage of Osx- and Ki67-double positive cells and the number of OSX positive cells per trabecular bone surface were quantified. **i** 8-week-old male *EP4*^*wt*^ and *EP4*_*Avil*_^*−/−*^ mice were injected with low dose (0.5 mg per kg per day) propranolol for 6 weeks. Representative images and quantitative analysis of the μCT images of femurs. Scale bar: 1 mm. *N* ≥ 5 per group. *P < 0.05, **P < 0.01 and N.S. means not significant. (Student *t-*test for **b**–**h**, ANOVA for **a**–**i**)

CREB signaling in the hypothalamus is crucial for the regulation of skeletal homeostasis^10^. To examine whether PGE2 could activate EP4 in sensory nerves through the ventromedial nucleus of the hypothalamus (VMH), we examined the effect of PGE2 on DRG neurons and the phosphorylation of CREB in the VMH of *EP4*_*Avil*_^*−/−*^ mice. Calcium imaging showed more illuminated DRG neurons in those pre-treated with PGE2 relative to vehicle-treated neurons, whereas DRG neuron activation was reduced significantly in *EP4*_*Avil*_^*−/−*^ mice with or without PGE2 pre-treatment (Fig. 6a). Western blot analysis of the hypothalamus showed that phosphorylation of CREB increased gradually and peaked at 6 h after injection (Supplementary Figure 12, A). Immunostaining of VMH sections showed that phosphorylation of CREB decreased significantly in *EP4*_*Avil*_^*−/−*^ mice relative to their WT littermates (Fig. 6b). PGE2 was then injected into *EP4*_*Avil*_^*−/−*^ mice and their WT littermates to further test whether the central regulation is sensory nerve-dependent. Immunostaining of hypothalamus sections showed CREB phosphorylation increased significantly in mouse VMH 6 h after injection, whereas, PGE2-induced CREB phosphorylation in VMH was abolished in *EP4*_*Avil*_^*−/−*^ mice (Fig. 6b). Then, EP1/3 and EP4 agonists were injected to examine whether EP4 receptor is responsible for PGE2-induced CREB signaling in VMH. EP1/3 agonist did not increase phosphorylation of CREB in VMH, whereas EP4 agonist significantly increased pCREB level relative to vehicle-treated mice (Supplementary Figure 12, B), indicating that EP4 in sensory nerves is specific for PGE2-induced CREB phosphorylation in VMH.

The activation of CREB signaling in the hypothalamus has been shown to suppress sympathetic tone^10,13^. Indeed, uncoupling protein 1 gene (*UCP1*) expression in adipose tissue and epinephrine concentrations in urine increased significantly in the *EP4*_*Avil*_^*−/−*^ mice and *COX2*_OC_^*−/−*^ mice relative to their WT littermates, indicating higher sympathetic tone in these two mouse models (Fig. 6c, d). Immunostaining of the femur sections showed small, flattened osteoblasts on the bone surface (Fig. 6e, f) and reduced Ki67 expression in osterix^+^ cells in both *COX2*_OC_^*−/−*^ and *EP4*_*Avil*_^*−/−*^ mice, indicating that increased sympathetic tone suppress osteoblastic activity (Fig. 6g, h). To confirm that the increased sympathetic activity leads to bone loss, we injected propranolol, a β2-adrenergic antagonist, into *EP4*_*Avil*_^*−/−*^ mice and *COX2*_OC_^*−/−*^ mice. Propranolol partially rescued bone phenotype of these two knockout mice (Fig. 6i and Supplementary Figure 13). These results indicate that sympathetic tone regulates osteoblast differentiation through EP4 activation of sensory nerve.

We also tested if PGE2 secretion is regulated by mechanical loading as mechanical loading has been shown to regulate bone homeostasis through central regulation of sympathetic tone^35,36^. Mechanical loading was applied to C57B/L6 mice and bone marrow PGE2 levels were measured. The result showed that PGE2 levels significantly increased in the loading group compared with the control group (Supplementary Figure 14A). COX2 expression in osteoblasts was examined in osteoblastic MC3T3-E1 cells cultured on high-extension silicon rubber dishes with applied compression force to mimic mechanical loads on bone. Western blot analysis showed that COX2 expression increased when the compression force applied (Supplementary Figure 14B). In addition, bone marrow PGE2 levels in *COX2*_OC_^*−/−*^ mice and their littermates with unloading by tail suspension were measured. The results showed that bone marrow PGE2 significantly decreased in wild-type mice in tail suspension conditions while no change was observed in *COX2*_OC_^*−/−*^ mice with (Supplementary Figure 14C). These results suggest that osteoblasts secrete PGE2 secretion in responsible to mechanical loading.

### PGE2 promotes skeletal regeneration through sensory nerves

**Fig. 7:**
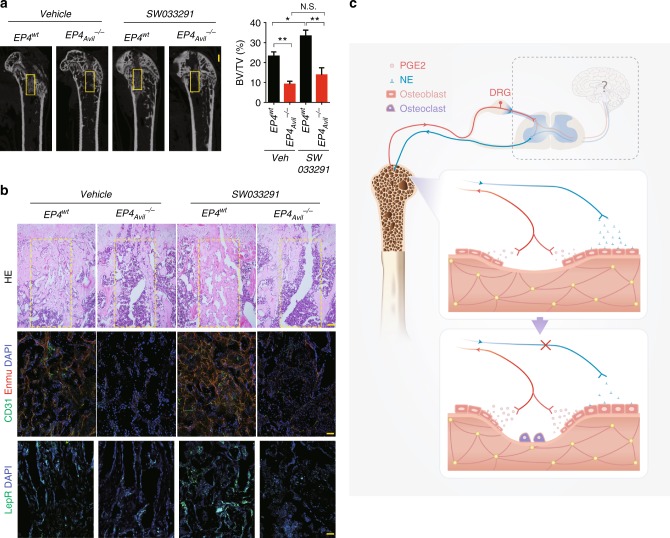
PGE2 promotes regeneration through sensory nerve. **a** μCT analysis of bone regeneration after femoral bone marrow ablation in 12-week-old *EP4*^*wt*^ and *EP4*_*Avil*_^*−/−*^ mice treated with 10 mg per kg per day SW033291 or vehicle. Scale bar: 1 mm. Selected areas for the measurements of bone volume (BV)/tissue volume (TV) were indicated with a yellow square. **b** Representative images of hematoxylin-eosin staining, double immunofluorescence analysis of CD31^+^ Emcn^+^ cells, and immunofluorescence analysis of Leptin receptor (LepR)^+^ cells in the regeneration area. Scale bar, 100 μm. **c** Graphic illustration of this study. When bone density decreases by osteoclast bone resorption, PGE2 secretion by osteoblastic cells increases at bone remodeling sites. PGE2 activates EP4 receptor at sensory nerve to tune down sympathetic tones for osteoblast differentiation at the bone remodeling microenvironment. The sensory nerve controlling process is likely a temporal-spatial precision action. *N* ≥ 5 per group. *P < 0.05, **P < 0.01 and N.S. means not significant. (ANOVA)

PGE2 has been reported recently to potentiate regeneration of multiple tissues^25^. To investigate whether PGE2 induces tissue regeneration through sensory nerves, we assessed whether PGE2 induces bone regeneration. *15-PGDH* inhibitor SW033291 was injected into *EP4*_*Avil*_^*−/−*^ and EP^*wt*^ mice that had undergone surgical ablation of trabecular bone to examine the effects of an increase in local PGE2 on bone regeneration. Elevation of local PGE2 boosted trabecular bone regeneration significantly in *EP4*^*wt*^ mice injected with SW033291 relative to vehicle-treated controls, as shown by μCT (Fig. 7a) and hematoxylin-eosin (HE) staining (Fig. 7b). However, the regeneration of trabecular bone by SW033291 was obstructed in *EP4*_*Avil*_^*−/−*^ mice (Fig. 7a, b). As known that CD31^hi^ Endomucin^hi^ type H vessel couples with active new bone formation^37,38^, we further evaluated its expression and found that the type H vessel growth was significantly increased in the regeneration area of *EP4*^*wt*^ mice treated with SW033291. However, type H vessels were almost undetectable in *EP4*_*Avil*_^*−/−*^ mice with or without injection of SW033291 (Fig. 7b). Thus, PGE2 stimulates bone regeneration through sensory nerves.

To further investigate whether PGE2 induces regeneration by sensory nerves of tissues other than bone, we performed partial hepatectomy in *TrkA*_*Avil*_^*−/−*^ mice and their WT littermates treated with SW033291 or vehicle. BrdU and Ki67 staining of liver sections with partial hepatectomy showed that the regeneration rate decreased significantly in *TrkA*_*Avil*_^*−/−*^ mice treated with SW033291 relative to their WT littermates (Supplementary Figure 15). Staining of CGRP in the regeneration areas confirmed that sensory innervation in the liver was significantly reduced in *TrkA*_*Avil*_^*−/−*^ mice. These results show that PGE2 induces bone regeneration dependent on sensory nerves, and that sensory nerves are likely involved in regeneration of various tissues.

## Discussion

As the largest organ, bone mechanically supports the body. Changes in bone can increase the risk of bone fracture. It is imperative to monitor changes in bone density to maintain bone homeostasis. We have found that PGE2 levels are elevated during decline in bone density in various animal models, including osteoporotic mice. The current study shows that sensory nerves sense bone density through the concentration of PGE2. The signals from PGE2, by bonding with EP4 in the sensory nerves, regulate sympathetic nerve activity for osteoblastic bone formation through the central nervous system. High sympathetic tone is known to stimulate osteoclastic bone resorption by increasing osteoblast secretion of *Rankl* (receptor activator of nuclear factor kappa-B ligand). Sensory nerve activated by PGE2 promotes osteoblast proliferation and differentiation by tuning down sympathetic nerve activity. Therefore, the sympathetic nerve can regulate both osteoclastogenesis for bone resorption and osteoblast differentiation for bone formation, depending on the levels of its tone. Interestingly, deletion of sensory nerves did not affect bone development but did reduce bone volume in adult mice. Importantly, sensory nerves regulate bone formation by osteoblast secretion of PGE2 in the bone remodeling microenvironment. Thus, the primary function of sensory nerves in bone is to maintain and protect bone homeostasis by sensing PGE2 in bone.

PTH and mechanical loading are known to stimulate the bone remodeling and TGFβ couples the process. These factors of bone remodeling have been reported to increase PGE2 levels in the bone marrow or cultured osteoblasts^39–42^, indicating that factors in regulation of bone remodeling also promotes osteoblast secretion of PGE2. Moreover, our data and previous report demonstrate that mechanical loading stimulates PGE2 levels in the skeletal system^35,36,43^. In osteoporotic conditions, the relative mechanical load per bone remodeling unit area increases, which promotes osteoblasts on the bone surface to secrete more PGE2 to stimulate sensory nerves. A recent study showed that an increase in PGE2 through inhibition of its degradation enzyme activity with a small molecule (SW033291) promotes tissue regeneration in various tissues, including liver, intestine, and hematopoietic cells in the bone marrow^25^. We too found that the increase in PGE2 caused by SW033291 promotes bone regeneration. Importantly, the effect on bone regeneration was eliminated when *EP4* in the sensory nerves was knocked out in *EP4*_*Avil*_^*−/−*^ mice. Similarly, liver regeneration was also diminished in *TrkA*_*Avil*_^*−/−*^ mice with or without injection of SW033291. It is documented that sensory and sympathetic nerves are both innervated in the liver^44^, and active neural sprouting was observed after partial hepatectomy, suggesting an essential role of sensory nerves in liver regeneration^45^. However, we observed liver regeneration rate was not altered in the *EP4*_*Avil*_^*−/−*^ mice in compare with their wild type littermates, neither does SW033291 treatment show any difference between these two groups, indicating that sensory nerve is indispensable in liver regeneration, but the mechanism is not through PGE2-EP4 signaling on sensory nerves. These results suggest that PGE2-EP4 signaling on sensory nerve seems to maintain the integrity of skeletal system specifically.

Clinical studies have reported decreased BMD in patients with long-term use of non-steroidal anti-inflammatory drugs, which inhibit COX2 activity to reduce the production of PGE2^23^. Evidence shows that PGE2 stimulates osteoblast differentiation by activating cAMP signaling through G protein-coupled EP4 receptor^18^. However, knockout of *EP4* receptor in osteoblasts with no skeletal phenotype indicates that PGE2-induced bone formation is not caused by direct signaling through osteoblasts. Our data show that deletion of *EP4* receptor in peripheral sensory nerves leads to decreased bone density, and that injection of PGE2 is no longer able to stimulate bone formation. It has been well documented that sympathetic tone was tuned down with PGE2 injection peripherally, and sympathetic nerve action on bone metabolism has been shown through hypothalamic regulation^12,13,46,47^. Indeed, we show that injection of PGE2 stimulated phosphorylation of CREB in the hypothalamus, which was inhibited by knockout of *EP4* in the peripheral sensory nerves (*EP4*_*Avil*_^*−/−*^), which is the evidence of CNS involvement. More than 10% of clinical patients with central nervous system injury were observed to have heterotopic ossification^48^. These findings show that sensory nerve signals from PGE2 in bone remodeling sites are circled back through regulation by sympathetic nerves. However, it is still unclear whether PGE2 signaling from the bone remodeling sites regulate sympathetic tone through DRG, hypothalamus or both and feedback regulation of sympathetic activity is spatially specific. Our data suggest that the feedback is likely involved both DRG and hypothalamus. It is documented that sensory signals from the viscera are carried to the central nervous system by spinal and cranial afferents^49^. Sensory afferents from visceral and somatic sensory nerves converge onto common second-order neurons within the spinal dorsal horn, and a subset of these neurons convey the convergent signals to the diencephalon via the anterolateral spinothalamic tract^50,51^. This viscerotopically organized pathway provides direct and relayed inputs to the hypothalamus and other central nervous locations^52,53^. Therefore, the similar pathway is possibly employed in the PGE2-induced CREB phosphorylation in the hypothalamus. Herein, sensory nerve regulation appears to involve precise temporal-spatial action controlled by sympathetic nerves in the bone remodeling microenvironment, and the central nervous system likely coordinates the activities of different tissues for energy and calcium metabolism of bone formation.

## Methods

### Mice and in vivo treatment

The *iDTR*^*fl/fl*^ and *Dentin matrix acidic phosphoprotein 1-Cre* (*DMP1-Cre*) mice were purchased from the Jackson Laboratory. The *Advillin-Cre* (*Avil-Cre*) mouse strain was kindly provided by Xingzhong Dong (The Johns Hopkins University). The *Osteocalcin-Cre* (*OC-cre*) mice were obtained from Thomas J. Clemens (The Johns Hopkins University). The *TrkA*^*fl/fl*^ mice were obtained from David D. Ginty (Harvard Medical School). The *COX2*^*fl/fl*^ mice were provided by Harvey Herschman (University of California, Los Angeles). The *EP4*^*fl/fl*^ mice were obtained from Brian L. Kelsall (National Institutes of Health). Heterozygous male *Avil-Cre* mice (female *Avil-Cre* mice were not used to breed in case for the leakage of *TrkA* protein into the eggs) were crossed with a *TrkA*^*fl/fl*^, *EP4*^*fl/fl*^, or *iDTR*^*fl/fl*^ mouse. The offspring were intercrossed to generate the following genotypes: wild type (referred as WT in the text), *Avil-Cre* (Cre recombinase expressed driven by Advillin promoter), *TrkA*^*fl/fl*^ (mice homozygous for *TrkA* flox allele are referred to as *TrkA*^*wt*^ in the text), *EP4*^*fl/fl*^ (referred to as *EP4*^*wt*^ in the text), *iDTR*^*fl/fl*^*, Avil-Cre::EP4*^*fl/fl*^ (conditional deletion of *EP4* receptor in Advillin lineage cells, referred to as *EP4*_*Avil*_^*−/−*^ in the text), *Avil-Cre::TrkA*^*fl/fl*^ (referred to as *TrkA*_*Avil*_^*−/−*^ in the text), and *Avil-Cre::iDTR*^*fl/+*^ mice (referred to as *iDTR*_*Avil*_^*+/−*^ in the text). Heterozygous *OC-Cre* or *DMP1-Cre* mice were crossed with a *COX2*^*fl/fl*^ mouse; the offspring were intercrossed to generate the following genotypes: *WT*, *OC-Cre, DMP1-Cre, COX2*^*fl/fl*^ (referred to as *COX2*^*wt*^ in the text), *OC-Cre::COX2* ^*fl/fl*^ (referred to as *COX2*_*OC*_^*−/−*^ in the text), and *DMP1-Cre::COX2* ^*fl/fl*^ (referred to as *COX2*_*DMP1*_^*−/−*^ in the text) mice. Heterozygous *OC-cre* mice were crossed with a *EP4*^*fl/fl*^ mouse, the offspring were intercrossed to generated the following genotypes: *WT* (referred as *EP4*^*fl/fl*^) and *OC-cre:: EP4*^*fl/fl*^ (conditional deletion of *EP4* receptor in osteocalcin lineage cells, referred to as *EP4*_*OC*_^*−/−*^ in the text). The genotypes of the mice were measured by PCR analyses of genomic DNA, which was extracted from mouse tails within the following primers: *Avil-Cre*: forward: CCCTGTTCACTGTGAGTAGG, Reverse: GCGATCCCTGAACATGTCCATC, *WT*:AGTATCTGGTAGGTGCTTCCAG; *OC-Cre*: forward: CAAATAGCCCTGGCAGATTC, Reverse: TGATACAAGGGACATCTTCC; *DMP1-Cre* forward: TTGCCTTTCTCTCCACAGGT, Reverse: CATGTCCATCAGGTTCTTGC; *EP4* loxP allele forward: TCTGTGAAGCGAGTCCTTAGGCT, Reverse: CGCACTCTCTCTCTCCCAAGGAA;*COX2* loxP allele forward: AATTACTGCTGAAGCCCACC, Reverse: GAATCTCCTAGAACTGACTGG; *TrkA* loxP allele forward: AACAGTTTTGAGCATTTTCTATTGTTT, Reverse: CAAAGAAAACAGAAGAAAAATAATAC; *iDTR* loxP allele forward: GCGAAGAGTTTGTCCTCAACC, Reverse: AAAGTCGCTCTGAGTTGTTAT. 8 to 12-week-old C57BL/6 female mice (Jackson Lab) were anesthetized and underwent bilateral OVX or a sham operation from back approach. The aged mice (12 months old) were purchased from The Jackson Laboratory. All animals were maintained at the animal facility of The Johns Hopkins University School of Medicine. All animal experimental protocols were complied with all relevant ethical regulations and approved by the Animal Care and Use Committee of The Johns Hopkins University, Baltimore, MD, USA. We obtained whole blood samples by cardiac puncture immediately after euthanasia. Serum was collected by centrifuge at 200 × *g* for 15min and stored at −80 °C before analyses. Femurs, tibias, and urine of the mice were also collected.

The drugs and compounds used in this study are as follows: diphtheria toxin (DTX, Sigma-Aldrich, D0564); PGE2 (Cayman Chemical, 14010); EP1/3 agonist (Cayman Chemical, 14810); EP4 agonist (Cayman Chemical, 10580); propranolol (Sigma-Aldrich, 1576005); norepinephrine (Sigma-Aldrich, A7257); and SW033291 (Selleck, S7900). Dosages and time courses are noted in the corresponding text and figure legends.

### Behavioral analysis

Pole tests and grip strength tests were performed to evaluate motor neural activity changes in *TrkA*_*Avil*_^*−/−*^, *EP4*_*Avil*_^*−/−*^, and *COX2*_*OC*_^*−/−*^ mice. All tests were performed between 10:00 and 16:00 during the lights on cycle. For the pole test, a 9-mm-diameter metric as 0.76-m metal rod wrapped with bandage gauze was used as the pole. The time for a mouse to turn and the total time for it to reach the base of the pole was recorded. Before the test, the mice were trained for three consecutive days, and each training session consisted of three test trials. For grip strength, neuromuscular strength was measured as maximum holding force generated by the mice (Biosed, USA). Mice were placed to grasp a metal grid with their forelimbs or hindlimbs. The tail was pulled gently, and the maximum holding force was recorded by the force transducer when the mice released their grasp on the grid. The peak holding strength was recorded digitally and displayed in grams.

### μCT analyses

The femurs were harvested from mice, and the soft tissue around the bone was removed, followed by fixation overnight using 4% paraformaldehyde. μCT analyses were performed by using a high-resolution μCT scanner (SkyScan, 1174). The voltage of the scanning procedure was 65 kv with a 153-μA current. The resolution was set to 8.7 μm per pixel. Reconstruction software (NRecon, v1.6, SkyScan), data analysis software (CTAn, v1.9, SkyScan), and 3D model visualization software (CTVol, v2.0, SkyScan) were used to analyze the diaphyseal cortical bone and the metaphyseal trabecular bone parameters of the femurs. We created cross-sectional images of the femur to perform 2D analyses of the cortical bone and 3D analyses of the trabecular bone. The region of interest (ROI) of the trabecular bone was drawn beginning from 5% of the femur length proximal to the distal metaphyseal growth plate and extending proximally for another 5% of the total femur length. The trabecular bone volume fraction (BV/TV), trabecular thickness (Tb. Th), trabecular number (Tb. N), and trabecular separation (Tb. Sp) were collected from the 3D analyses data and used to represent the trabecular bone parameters. The cortical bone ROI was drawn beginning from 20% of femur length proximal to distal metaphyseal growth plate and extending proximally to another 10% of the total femur length. The cortical thickness (Ct. Th), periosteal perimeter (Ps. Pm), and endosteal perimeter (Es. Pm) were collected from the 2D analyses data and used to represent the cortical bone parameters.

### Immunohistochemistry, immunofluorescence and histomorphometry

The femurs were collected and fixed in 4% paraformaldehyde overnight and decalcified by using 10% EDTA (pH, 7.4) (Amresco, 0105) for 21 days. The samples were then dehydrated with 30% sucrose for 24 h and embedded in paraffin or optimal cutting temperature compound (Sakura Finetek). We prepared 4-μm-thick coronal-oriented sections of the femur for hematoxylin and eosin staining. The femurs were fixed for 4 h with 4% paraformaldehyde at 4°C and then decalcified at 4°C using 0.5M EDTA (pH, 7.4) for 24 h with constant shaking. The samples were dehydrated in 20% sucrose and 2% polyvinylpyrrolidone (PVP) solution for 24 h and embedded in 8% gelatin (Sigma-Aldrich, G1890) in the presence of 20% sucrose and 2% PVP. Forty-μm-thick coronal-oriented sections of the femurs were obtained. For brain section preparation, the whole brain was collected from euthanized mice and fixed with 4% paraformaldehyde for 30 mins. Then, the tissue was dehydrated with 20% sucrose for 24 h and sectioned.

Immunostaining was performed using standard protocol. Briefly, the sections were incubated with primary antibodies to mouse osterix (Abcam, ab22552, 1:600), osteocalcin (Takara Bio, M173, 1:200), CD31 (Abcam, ab28326, 1:50), endomucin (Santa Cruz, V.7C7, 1:50), Ki67 (Abcam, ab16667, 1:100), CGRP (Abcam, ab81887, 1:100), COX2 (Abcam, ab15191, 1:100), EP4 (Abcam, ab92763, 1:10), CREB (Cell Signaling Technology, 9197, 1:100), p-CREB (Abcam, ab32096, 1:100), NF200 (Millipore, AB5539, 1:500), TrkA (R& D systems, AF1056, 1:1000) and IB4 (Thermo Fisher Scientific, I21411) overnight at 4 °C. A horseradish peroxidase–streptavidin detection kit (Dako) was used in immunohistochemical procedures to detect immuno-activity, followed by counterstaining with hematoxylin (Dako, S3309). Fluorescence-conjugated secondary antibodies were used in immunofluorescent procedures to detect fluorescent signals after counterstaining with DAPI (Vector, H-1200). We used a Zeiss LSM 780 confocal microscope or an Olympus BX51 microscope for sample image capturing. A BrdU staining kit (Thermo Fisher Scientific, 8800-6599-45) was used to perform the BrdU immunostaining procedure. Quantitative histomorphometric analysis was performed by using OsteoMeasure XP Software (OsteoMetric) in a blinded fashion.

A double-labeling procedure was performed to measure dynamic bone formation. Briefly, we injected 0.1% calcein (Sigma-Aldrich, C0875) in phosphate-buffered saline at a concentration of 10mg per kg into the mice subcutaneously 7 days and 1 day before sacrifice. The double-labeling images of undecalcified bone slices were captured using a fluorescence microscope. We analyzed trabecular bone formation in four randomly selected visual fields in the distal metaphyseal area of the femur.

### Quantitative real-time polymerase reaction chain (qPCR)

Total RNA was purified from cells in culture or tissues using TRIzol (Invitrogen, 15596026), following the manufacturer’s protocol. We performed qPCR using the Taq SYBR Green Power PCR Master Mix (Invitrogen, A25777) on a CFX Connect instrument (Bio-Rad); *Gapdh* amplification was used as an internal control. Dissociation curves analysis was performed for every experiment. Sequences of the primers used for each gene are as listed: *EP4* forward: CGGTTCCGAGACAGCAAA, Reverse: CGGTTCGATCTAGGAATGG. *UCP1* forward: CTTTGCCTCACTCAGGATTGG, Reverse: ACTGCCACACCTCCAGTCATT. *TrkA*: AGAGTGGCCTCCGCTTTGT, Reverse: CGCATTGGAGGACAGATTCA. *Gapdh* forward: ATGTGTCCGTCGTGGATCTGA, Reverse: ATGCCTGCTTCACCACCTTCTT.

### Bone marrow supernatant collection

We euthanized mice, cut two ends of the tibia, and centrifuged the samples for 15 min at 800 × *g* at 4 °C to obtain bone marrow supernatants, which were stored at –80 °C until ELISA.

### ELISA and western blot testing

PGE2 concentrations in the serum and bone marrow were determined by PGE2 ELISA kit (Cayman Chemical, 514010) according to the manufacturer’s protocol. Mice serum was collected as described above. We also performed osteocalcin and CTX ELISA of serum using a mouse osteocalcin enzyme immunoassay kit (Biomedical Technologies, BT-470) and a RatLaps enzyme immunoassay kit (Immunodiagnostic Systems, AC06F1).

Western blot analysis was conducted on the basis of the protein lysates from the hypothalamus of mice or cultured cell line. The lysates were centrifuged; the supernatants were collected and separated by SDS-PAGE PAGE (sodium dodecyl sulfate-polyacrylamide gel electrophoresis) and then blotted on the polyvinylidene fluoride membrane (Bio-Rad Laboratories). Specific antibodies were applied for incubation, and the proteins were detected by using an enhanced chemiluminescence kit (Amersham Bioscience, RNP2108). The antibodies used for western blotting were HTR2C (Abcam, ab197776, 1:500), CREB (Cell Signaling Technology, 9197, 1:500), p-CREB (Abcam, ab32096, 1:500), COX2 (Abcam, ab15191, 1:1000), and GAPDH (Cell Signaling Technology, 5174, 1:1000).

### DRG culture and calcium imaging

DRGs from the L2-L5 spinal levels of 4-week-old mice were isolated in cold DMEM/F12 medium (Invitrogen, 11039-021) and then treated with collagenase type A (Roche, 10103578001) at 37 °C. After trituration and centrifugation, cells were resuspended and plated on glass coverslips coated with ploy-D-lysine and laminin. The cells were then cultured in an incubator at 37 °C. Primary isolated DRG neurons were loaded with Fura-2-acetomethoxyl ester (Molecular Probes) for 45 min in the dark at room temperature. The cells were imaged at 340 and 380 nm after immersion in calcium-free buffer for 1 min.

### In vitro and in vivo mechanical loading assays

In vitro-mechanical stretching assay: Osteoblastic MC3T3 cells (stored in our lab) were plated on the home-made high-extension silicone rubber dishes with 4% and 12% morphological change for 24 h and starved for 4 h. The high-extension silicone rubber dishes were fixed on the home-made mechanical stretching machine and compress the cells with different force reflected as 4% and 12% morphology change of the dishes. After 24 h, the cells lysate was harvested for western blot assays.

In vivo-treadmill assay: C57BL/6 mice were trained for 5 days on treadmill with the protocol of 8m/min, 5° uphill for 10 min per day. Then, we followed the protocol of 14m/min, 14° uphill for 20min per day for formal tests. After 2 weeks, serum and bone marrow were harvested for ELISA assays.

### Bone and liver regeneration models

Mice underwent general anesthesia. The bone regeneration model was established as described below. A longitudinal incision was made on each knee to expose the femoral condyle by patella dislocation. Then, a hole was made at the intercondylar notch of the femur by using a dental drill. A 0.6-mm-diameter Kirschner wire was placed from the proximal end of the femur to confirm marrow ablation by radiography. The dislocated patella was reposed, and the skin was sutured after removal of the Kirschner wire. Bone samples were harvested 7 days after bone marrow ablation, as described above.

Partial hepatectomy was used for the liver regeneration model. 10- to 12-week-old *TrkA*_*Avil*_^*F592A*^ male mice were anesthetized, as described above. A partial (two-thirds) hepatectomy was performed by resecting the median and left lateral hepatic lobes^54^. The remnant livers were harvested after mice sacrifice. SW033291 was dissolved in a vehicle of 10% ethanol, 5% Cremophor EL, and 85% dextrose 5% in water.

### Statistics

All data analyses were performed using SPSS, version 15.0, software (IBM Corp.). Data are presented as means ± standard error of mean (SEM). For comparisons between two groups, we used two-tailed Student *t*-tests. For comparisons among multiple groups, we used one-way ANOVA. All inclusion/exclusion criteria were pre-established, and no samples or animals were excluded from the analysis. No statistical method was used to predetermine the sample size. The experiments were randomized. The investigators were not blinded to allocation during experiments and outcome assessment. All relevant data are available from the authors.

### Reporting summary

Further information on experimental design is available in the Nature Research Reporting Summary linked to this article.

## Supplementary information

Supplementary Information

Reporting Summary

## Data Availability

We confirm that all relevant data are available from the authors.
