# Supplementary material for: Prostaglandin E2 mediates sensory nerve regulation of bone homeostasis
Source: Nat Commun. 2019 Jan 14;10:181. doi: 10.1038/s41467-018-08097-7 (PMC6331599; doi:10.1038/s41467-018-08097-7)
Supplement: Supplementary file 1 — Supplementary Information [file 41467_2018_8097_MOESM1_ESM.pdf]

Prostaglandin E2 Mediates Sensory Nerve Regulation of Bone Homeostasis

Chen et al.

Supplementary Figure 1

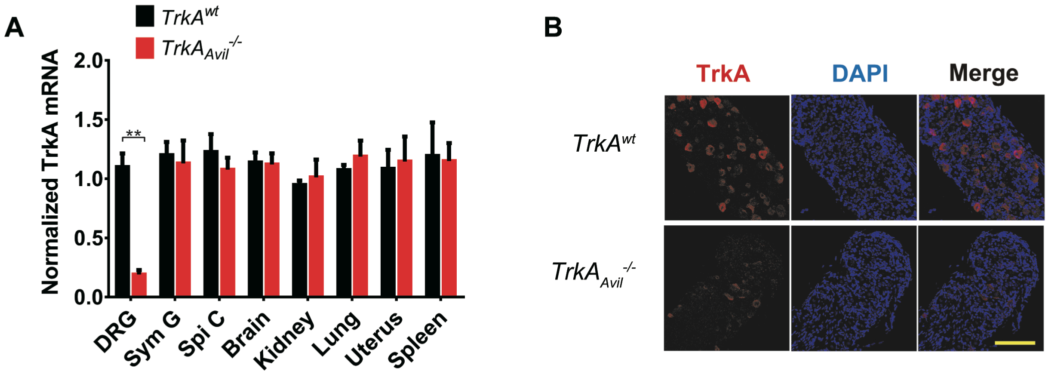

Supplementary Figure 1. (**A**) Determination of targeted-knockout efficiency of *TrkA* gene in $TrkA_{Avil}^{-/-}$ mice by qPCR of mRNA isolated from different type tissue, including dorsal root gangolion (DRG), sympathetic gangolion (Sym G), spinal cord (Spi C), kidney, lung, uterus and spleen. (**B**) TrkA (Red) and NeuN (Green) double-immunofluorescence images of the femur to confirm the TrkA expression on DRG neurons was ablated in $TrkA_{Avil}^{-/-}$ mice. Scale bar, 20 µm. N=3 per group. $^{**}P$<0.01 (Student *t* test).

Supplementary Figure 2

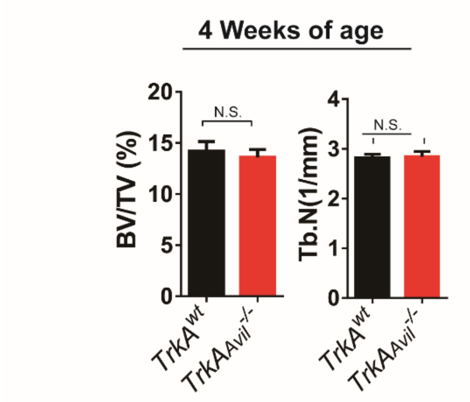

Supplementary Figure 2. Quantative analysis of trabecular bone fraction (Tb. BV/TV) and trabecular number (Tb. N) in 4-week-old $TrkA^{wt}$ and $TrkA_{Avil}^{-/-}$ mice femur. N=5 per group. N.S. means not significant (Student *t* test).

Supplementary Figure 3

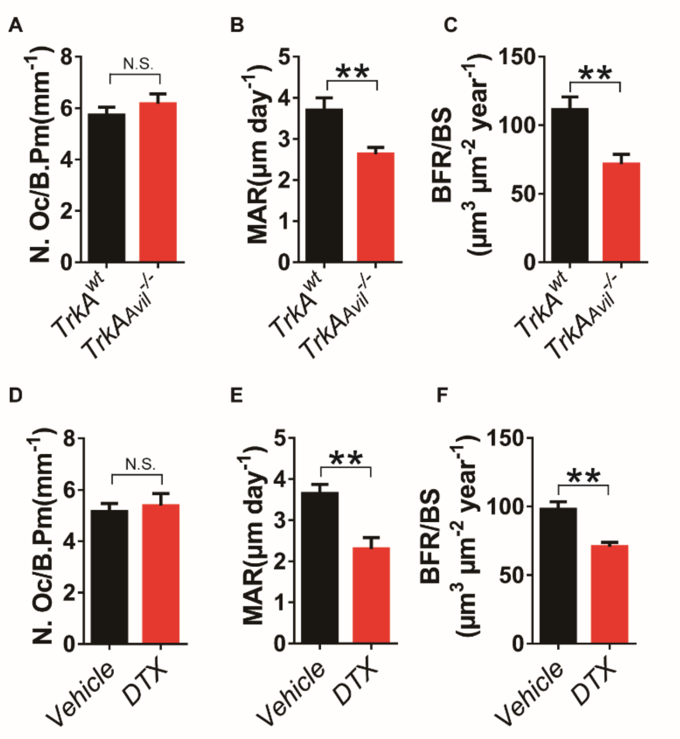

Supplementary Figure 3. (**A**) Histomorphological analysis of osteoclast (N.Oc/B.Pm) numbers on the trabecular bone surface of vertebrae of 12-week-old $TrkA^{wt}$ and $TrkA_{Avil}^{-/-}$ mice. (**B, C**) Quantification of mineral apposition rate and bone formation rate in 12-week-old $TrkA^{wt}$ and $TrkA_{Avil}^{-/-}$ mice vertebrae. (**D**) Histomorphological analysis of osteoclast (N.Oc/B.Pm) numbers on the trabecular bone surface of vertebrae of $iDTR_{Avil}^{+/-}$ mice injected with vehicle or 1 ug per kg per day DTX. (**E, F**) Quantification of mineral apposition rate and bone formation rate of the vertebral bone in $iDTR_{Avil}^{+/-}$ mice injected with vehicle or DTX. N≥ 5 per group. *P<0.05, **P<0.01 and N.S. means not significant. (Student t test).

Supplementary Figure 4

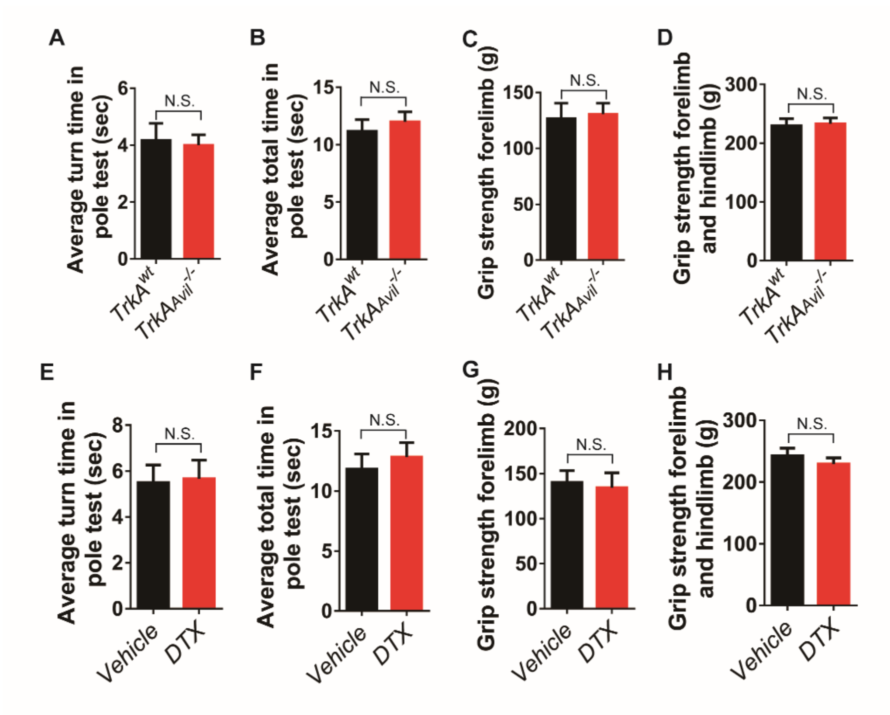

Supplementary Figure 4. Behavior test of $TrkA_{Avil}^{wt}$, $TrkA_{Avil}^{-/-}$ and $iDTR_{Avil}^{+/-}$ mice injected with vehicle or DTX. (**A, B**) The average turn time and the average total time to complete the pole test for $TrkA_{Avil}^{wt}$ and $TrkA_{Avil}^{-/-}$ mice. (**C, D**) Grip strength of the forelimbs and hindlimbs of $TrkA_{Avil}^{wt}$ and $TrkA_{Avil}^{-/-}$ mice. (**E, F**) The average turn time and the average total time to complete the pole test for $iDTR_{Avil}^{+/-}$ mice injected with vehicle or DTX. (**G, H**) Grip strength of the forelimbs and hindlimbs of $iDTR_{Avil}^{+/-}$ mice injected with vehicle or DTX. (N=6 per group. No significant differences per Student *t* tests).

Supplementary Figure 5

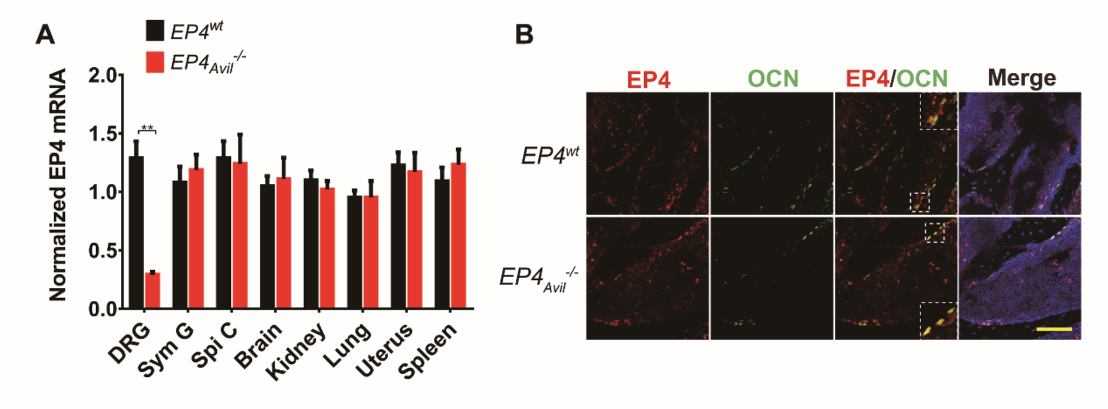

Supplementary Figure 5. (**A**) Determination of targeted-knockout efficiency of *EP4* gene in $EP4_{Avil}^{-/-}$ mice by qPCR of mRNA isolated from different types of tissue, including dorsal root gangolion (DRG), sympathetic ganglion (Sym G), spinal cord (Spi C), kidney, lung, uterus, and spleen. (**B**) EP4 (red) and OCN (green) double-immunofluorescence images of the femur confirm that EP4 expression of osteoblasts was unaffected in $EP4_{Avil}^{-/-}$ mice. Scale bar, 50 µm. N=3 per group. ***P*<0.01 (Student *t* test).

Supplementary Figure 6

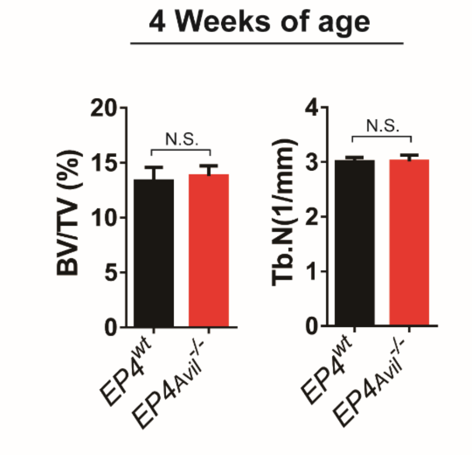

Supplementary Figure 6. Quantative analysis of trabecular bone fraction (Tb. BV/TV) and trabecular number (Tb. N) in 4-week-old $EP4^{wt}$ and $EP4_{Avil}^{-/-}$ mice femur. N=5 per group. N.S. means not significant (Student *t* test).

Supplementary Figure 7

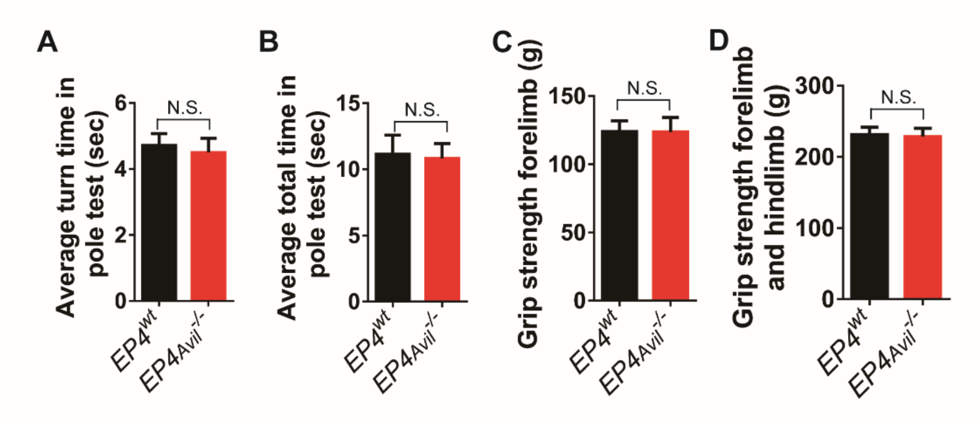

Supplementary Figure 7. The average turn time (**A**) and average total time (**B**) to complete the pole test for $EP4^{wt}$ and $EP4_{Avil}^{-/-}$ mice. Grip strength of the forelimbs (**C**) and hindlimbs (**D**) of $EP4^{wt}$ and $EP4_{Avil}^{-/-}$ mice. (N≥ 7 per group. No significant differences per Student *t* tests).

Supplementary Figure 8

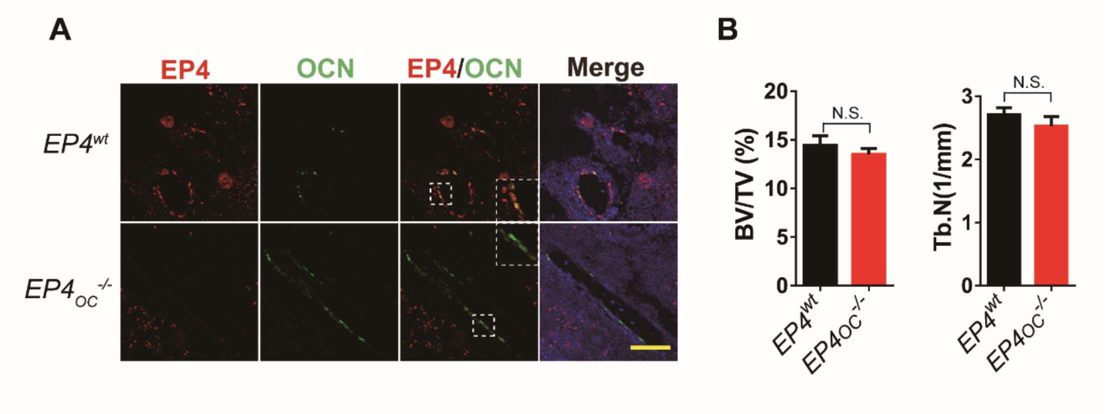

Supplementary Figure 8. (**A**) EP4 (red) and OCN (green) double-immunofluorescence images of the femur to confirm that the EP4 was no longer expressed in the osteoblasts of $EP4_{OC}^{-/-}$ mice. Scale bar, 50 μm. (**B**) Quantitative analysis of trabecular bone fraction (Tb. BV/TV) and trabecular number (Tb. N) of the femurs from $EP4^{wt}$ and $EP4_{OC}^{-/-}$mice. (N=5 per group. No significant differences per Student *t* tests).

Supplementary Figure 9

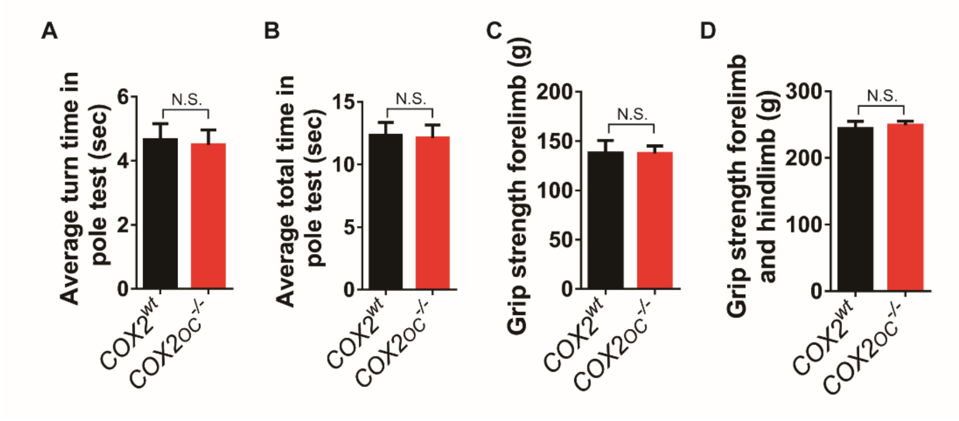

Supplementary Figure 9. Behavior test of *COX2*^{wt} and *COX2*$_{OC}^{-/-}$ mice. (**A, B**) The average turn time and the average total time to complete the pole test for *COX2*^{wt} and *COX2*$_{OC}^{-/-}$ mice. (**C, D**) Grip strength of the forelimbs and hindlimbs of *COX2*^{wt} and *COX2*$_{OC}^{-/-}$ mice. (N$\geq$ 6 per group. No significant differences on Student *t* tests).

Supplementary Figure 10

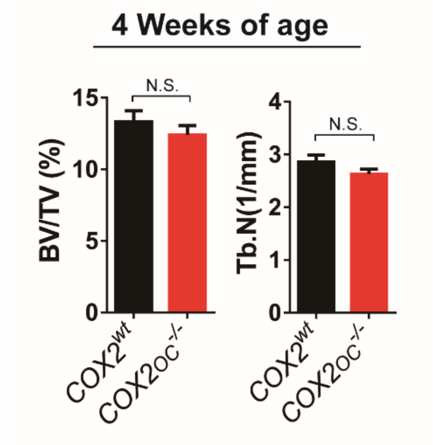

Supplementary Figure 10. Quantative analysis of trabecular bone fraction (Tb. BV/TV) and trabecular number (Tb. N) in 4-week-old *COX2*^{wt} and *COX2*$_{OC}^{-/-}$ mice femur. N=5 per group. N.S. means not significant (Student *t* test).

Supplementary Figure 11

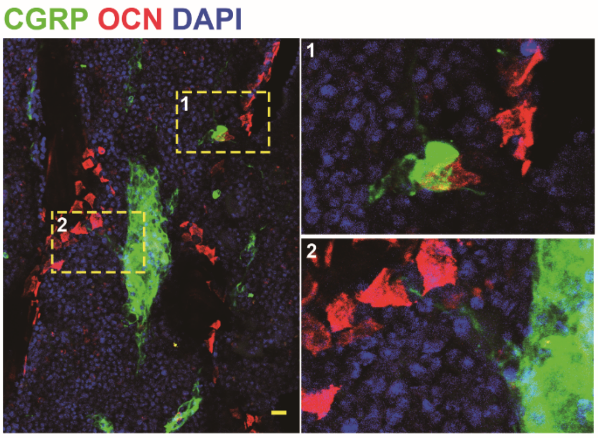

Supplementary Figure 11. Double-immunofluorescence images of femoral bone sections of 12-week-old C57BL/6 mice using antibodies against CGRP (green) and OCN (red). DAPI stains nuclei blue. Scale bar, 20 μm.

Supplementary Figure 12

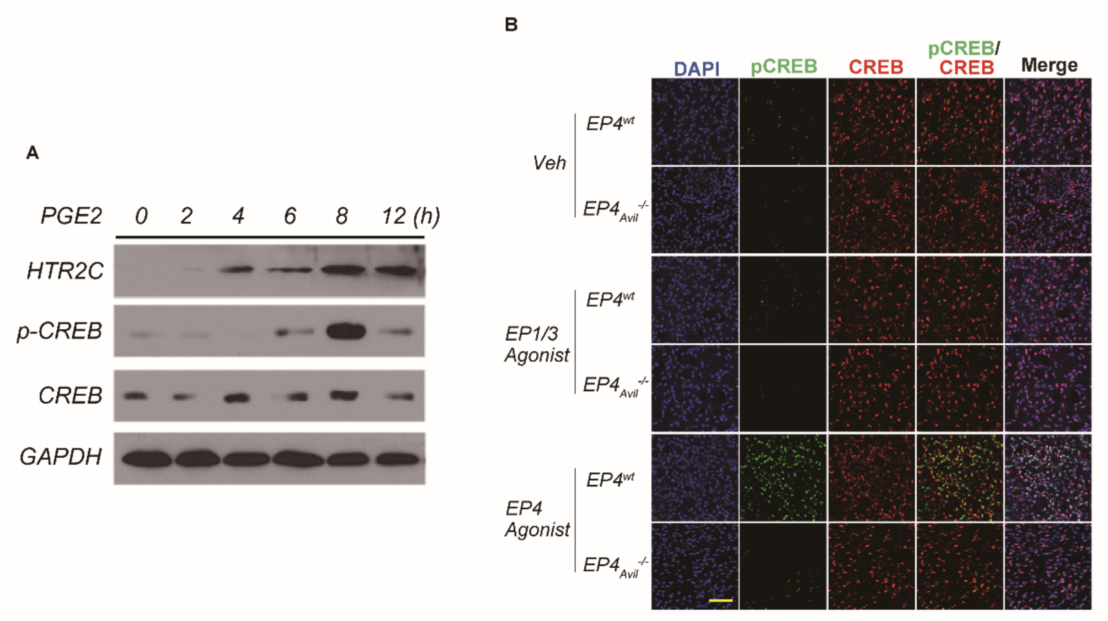

Supplementary Figure 12. (**A**) Western blot analysis of HTR2C and p-CREB in hypothalamus tissue from 12-week-old mice after PGE2 or vehicle injection at different periods. (**B**) Double-immunofluorescence images of hypothalamus tissue sections from C57BL/6 mice with vehicle, EP1/3 agonist (200 ug per kg) or EP4 agonist (200 ug per kg) treatment for 6 hours using antibodies against CREB (red) and p-CREB (green). DAPI stains nuclei blue. Scale bar, 50 μm.

Supplementary Figure 13

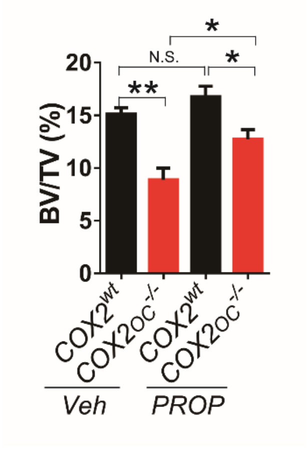

Supplementary Figure 13. 8-week-old male *COX2*^{wt} and *COX2*$_{OC}^{-/-}$ mice were injected with low does (0.5 mg per kg per day) propranolol for 6 weeks. Representative images and quantitative analysis of the μCT images of femurs. N=5 per group. **P*<0.05, ***P*<0.01 and N.S. means not significant. (ANOVA).

Supplementary Figure 14

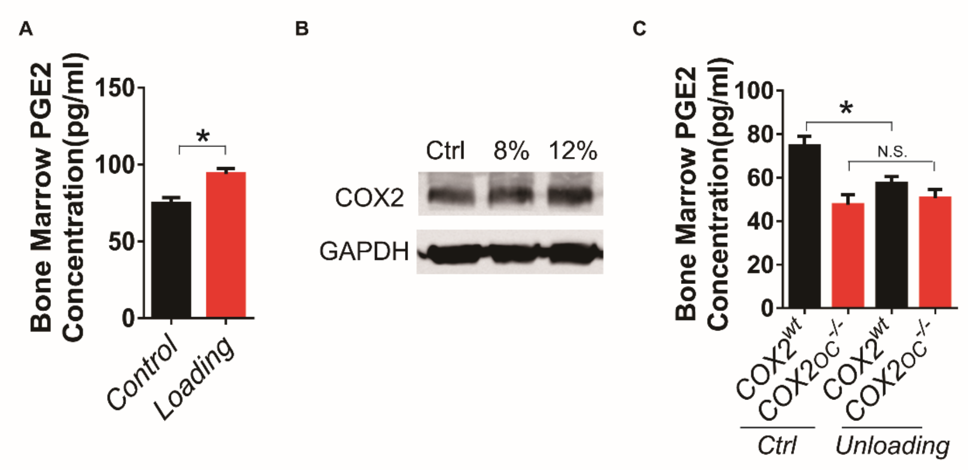

Supplementary Figure 14. (**A**) ELISA evaluation of bone marrow PGE2 concentrations(pg per ml) in control and loading applied mice. (B) Western blot analysis of COX2 expression change in osteoblastic MC3T3 cells treated with different loading force. (C) ELISA evaluation of bone marrow PGE2 concentrations(pg per ml) in control and unloading treated COX2$_{OC}^{-/-}$ mice. N=4 per group. **P*<0.05, ***P*<0.01 and N.S. means not significant. (Student *t* test and ANOVA).

Supplementary Figure 15

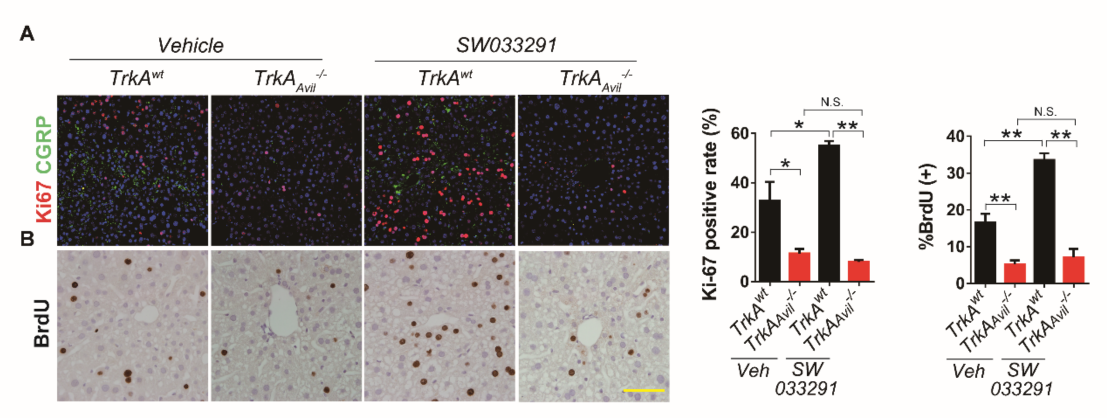

Supplementary Figure 15. (**A**) Double-immunofluorescence images of liver tissue sections from *TrkA*^{wt} and *TrkA*$_{Avil}^{-/-}$ mice with vehicle or SW033291 treatment using antibodies against and Ki67 (red) and CGRP (green). DAPI stains nuclei blue. Percentages of the Ki67^{+} cells are shown in the right panel. Scale bar, 100 µm. (**B**) BrdU staining images of liver tissue sections from *TrkA*^{wt} and *TrkA*$_{Avil}^{-/-}$ mice with vehicle or SW033291 treatment. Scale bar, 100 mm. Percentages of the BrdU^{+} cells are shown in the right panel. N=4 per group. **P*<0.05, ***P*<0.01 and N.S. means not significant. (ANOVA).
